# 1,1′-Diphenyl-3,3′-(*p*-phenyl­enedicarbon­yl)dithio­urea

**DOI:** 10.1107/S1600536809055834

**Published:** 2010-01-09

**Authors:** Wong W. Hung, Ibrahim N. Hassan, Bohari M. Yamin, Mohammad B. Kassim

**Affiliations:** a School of Chemical Sciences and Food Technology, Faculty of Science and Technology, Universiti Kebangsaan Malaysia, 43600 Bangi Selangor, Malaysia

## Abstract

The mol­ecule of the title compound, C_22_H_18_N_4_O_2_S_2_, lies across a crystallographic inversion centre. The central benzene ring forms dihedral angles of 29.39 (9) and 79.11 (12)°, respectively, with the thio­urea unit and the terminal phenyl ring. Intra­molecular N—H⋯O hydrogen bonds generate two *S*(6) ring motifs. In the crystal, mol­ecules are linked into chains along [1
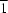
0] by inter­molecular N—H⋯S hydrogen bonds.

## Related literature

For general background and crystal structures of thio­urea derivatives, see: Dong *et al.* (2006 ▶); Hassan *et al.* (2008 ▶); Yamin & Hassan (2004 ▶). For bond-length data, see: Allen *et al.* (1987 ▶).
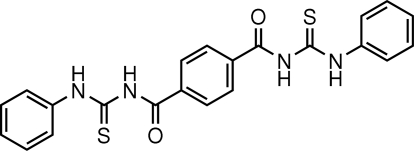


## Experimental

### 

#### Crystal data

C_22_H_18_N_4_O_2_S_2_
*M* *_r_* = 434.52
Triclinic, 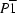

*a* = 5.769 (2) Å
*b* = 7.919 (3) Å
*c* = 11.534 (4) Å
α = 75.961 (10)°
β = 87.000 (8)°
γ = 89.861 (8)°
*V* = 510.5 (3) Å^3^
*Z* = 1
Mo *K*α radiation
μ = 0.29 mm^−1^
*T* = 273 K
0.23 × 0.11 × 0.05 mm

#### Data collection

Bruker SMART APEX CCD area-detector diffractometer
Absorption correction: multi-scan (*SADABS*; Bruker, 2000 ▶) *T*_min_ = 0.937, *T*_max_ = 0.986
5484 measured reflections
1809 independent reflections
1503 reflections with *I* > 2σ(*I*)
*R*_int_ = 0.030

#### Refinement

*R*[*F* ^2^ > 2σ(*F* ^2^)] = 0.051
*wR*(*F* ^2^) = 0.113
*S* = 1.13
1809 reflections
136 parameters
H-atom parameters constrained
Δρ_max_ = 0.25 e Å^−3^
Δρ_min_ = −0.16 e Å^−3^

### 

Data collection: *SMART* (Bruker, 2000 ▶); cell refinement: *SAINT* (Bruker, 2000 ▶); data reduction: *SAINT* program(s) used to solve structure: *SHELXS97* (Sheldrick, 2008 ▶); program(s) used to refine structure: *SHELXL97* (Sheldrick, 2008 ▶); molecular graphics: *SHELXTL* (Sheldrick, 2008 ▶); software used to prepare material for publication: *SHELXTL* and *PLATON* (Spek, 2009 ▶).

## Supplementary Material

Crystal structure: contains datablocks global, I. DOI: 10.1107/S1600536809055834/ci5010sup1.cif

Structure factors: contains datablocks I. DOI: 10.1107/S1600536809055834/ci5010Isup2.hkl

Additional supplementary materials:  crystallographic information; 3D view; checkCIF report

## Figures and Tables

**Table 1:** Hydrogen-bond geometry (Å, °)

*D*—H⋯*A*	*D*—H	H⋯*A*	*D*⋯*A*	*D*—H⋯*A*
N1—H1*A*⋯O1	0.86	1.94	2.644 (3)	138
N2—H2*A*⋯S1^i^	0.86	2.62	3.446 (3)	160

Symmetry code: (i) 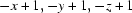.

**Fig. 1.**
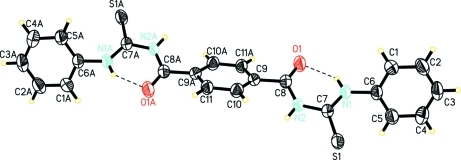
The molecular structure of the title compound, with displacement ellipsoids drawn at the 50% probability level. Atoms labelled with the suffix A are generated by the symmetry operation (2-x, -y, 1-z).

**Fig. 2.**
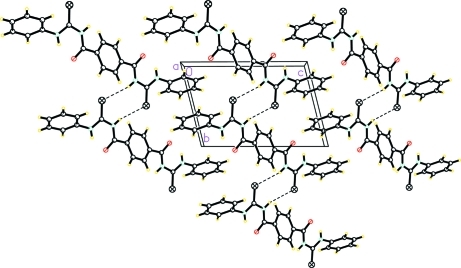
Crystal packing of of the title compound, viewed down the a axis. Hydrogen bonds are shown as dashed lines.

## supplementary crystallographic information

### Comment

The asymmetric unit of the title compound contains one-half of the molecule the other half being generated by the crystallographic inversion centre (Fig. 1). The thiourea fragment (S1/O1/N1/N2/C6/C7/C8) is planar, with atom C8 has the maximum deviation of 0.038 (2)Å from the mean plane. The dihedral angle between the central bridging benzene ring and the thiourea unit is 29.39 (9)° and that between the two benzene rings is 79.11 (12)°. The carbonyl and N-H groups are involved in intramolecular N—H···O hydrogen bonding resulting in the formation of two six-membered rings viz. C7/N2/C8/O1/H1A/N1 and C7A/N2A/C8A/O1A/H1AA/N1A.  The C═O bond length of 1.220 (3) Å is longer than the average C═O bond length (1.200 Å). These features are similar to those observed in the structure of *N*-benzoyl-*N*'-(3-pyridyl)thiourea (Dong *et al.*, 2006). Bond lengths are in normal ranges (Allen *et al.*, 1987).

In the crystal structure, intermolecular N—H···S hydrogen bonds (Table 1) link the molecules into a chain along the [110] (Fig 2).

### Experimental

The title compound was synthesized according to a previously reported method (Hassan *et al.*, 2008) with some modification. Terephthaloyl chloride (1.015 g, 0.5 mmol) was added to ammonium thiocyanate (0.770 g, 1 mmol) in tetrahydrofuran and the contents were stirred for 10 min. The precipitated ammonium chloride was removed by filtration and then aniline (1.0 ml, 1 mmol) in methanol was added dropwise. The mixture was refluxed for 5 h and the black coloured solution was dried using a evaporator before it was washed with cool methanol. Yellow crystals of the title compound were obtained by recrystallization from DMF.

### Refinement

H atoms were positioned geometrically [N-H = 0.86 Å and C-H = 0.93Å] and allowed to ride on their parent atoms, with *U*_iso_(H) = 1.2*U*_eq_(C,N).

### Crystal data

**Table:**

C_22_H_18_N_4_O_2_S_2_	*Z* = 1
*M**_r_* = 434.52	*F*(000) = 226
Triclinic, *P*1	*D*_x_ = 1.413 Mg m^−^^3^
Hall symbol: -P 1	Mo *K*α radiation, λ = 0.71073 Å
*a* = 5.769 (2) Å	Cell parameters from 1272 reflections
*b* = 7.919 (3) Å	θ = 1.8–25.0°
*c* = 11.534 (4) Å	µ = 0.29 mm^−^^1^
α = 75.961 (10)°	*T* = 273 K
β = 87.000 (8)°	Plate, yellow
γ = 89.861 (8)°	0.23 × 0.11 × 0.05 mm
*V* = 510.5 (3) Å^3^	

### Data collection

**Table:**

Bruker SMART APEX CCD area-detector diffractometer	1809 independent reflections
Radiation source: fine-focus sealed tube	1503 reflections with *I* > 2σ(*I*)
graphite	*R*_int_ = 0.030
ω scan	θ_max_ = 25.0°, θ_min_ = 1.8°
Absorption correction: multi-scan (*SADABS*; Bruker, 2000)	*h* = −6→6
*T*_min_ = 0.937, *T*_max_ = 0.986	*k* = −9→9
5484 measured reflections	*l* = −13→13

### Refinement

**Table:**

Refinement on *F*^2^	Primary atom site location: structure-invariant direct methods
Least-squares matrix: full	Secondary atom site location: difference Fourier map
*R*[*F*^2^ > 2σ(*F*^2^)] = 0.051	Hydrogen site location: inferred from neighbouring sites
*wR*(*F*^2^) = 0.113	H-atom parameters constrained
*S* = 1.13	*w* = 1/[σ^2^(*F*_o_^2^) + (0.0449*P*)^2^ + 0.1485*P*] where *P* = (*F*_o_^2^ + 2*F*_c_^2^)/3
1809 reflections	(Δ/σ)_max_ = 0.001
136 parameters	Δρ_max_ = 0.25 e Å^−^^3^
0 restraints	Δρ_min_ = −0.16 e Å^−^^3^

### Special details

**Table:**

Geometry. All e.s.d.'s (except the e.s.d. in the dihedral angle between two l.s. planes) are estimated using the full covariance matrix. The cell e.s.d.'s are taken into account individually in the estimation of e.s.d.'s in distances, angles and torsion angles; correlations between e.s.d.'s in cell parameters are only used when they are defined by crystal symmetry. An approximate (isotropic) treatment of cell e.s.d.'s is used for estimating e.s.d.'s involving l.s. planes.
Refinement. Refinement of *F*^2^ against ALL reflections. The weighted *R*-factor *wR* and goodness of fit *S* are based on *F*^2^, conventional *R*-factors *R* are based on *F*, with *F* set to zero for negative *F*^2^. The threshold expression of *F*^2^ > σ(*F*^2^) is used only for calculating *R*-factors(gt) *etc*. and is not relevant to the choice of reflections for refinement. *R*-factors based on *F*^2^ are statistically about twice as large as those based on *F*, and *R*- factors based on ALL data will be even larger.

### Fractional atomic coordinates and isotropic or equivalent isotropic displacement parameters (Å^2^)

**Table:**

	*x*	*y*	*z*	*U*_iso_*/*U*_eq_	
S1	0.32753 (13)	0.52539 (9)	0.65718 (6)	0.0563 (3)	
N1	0.2814 (3)	0.2027 (3)	0.79479 (17)	0.0451 (5)	
H1A	0.3202	0.0953	0.8057	0.054*	
N2	0.5449 (3)	0.2421 (2)	0.63257 (16)	0.0402 (5)	
H2A	0.6037	0.3126	0.5690	0.048*	
O1	0.5453 (3)	−0.0411 (2)	0.73721 (17)	0.0647 (6)	
C1	0.1594 (5)	0.1854 (3)	1.0006 (2)	0.0506 (7)	
H1B	0.2946	0.1251	1.0236	0.061*	
C2	−0.0015 (5)	0.2172 (4)	1.0865 (2)	0.0580 (8)	
H2B	0.0274	0.1796	1.1672	0.070*	
C3	−0.2010 (5)	0.3030 (4)	1.0531 (3)	0.0570 (8)	
H3A	−0.3086	0.3232	1.1111	0.068*	
C4	−0.2439 (5)	0.3599 (4)	0.9342 (3)	0.0582 (8)	
H4A	−0.3807	0.4184	0.9119	0.070*	
C5	−0.0854 (4)	0.3310 (3)	0.8470 (2)	0.0504 (7)	
H5A	−0.1148	0.3696	0.7664	0.060*	
C6	0.1173 (4)	0.2439 (3)	0.8812 (2)	0.0413 (6)	
C7	0.3798 (4)	0.3128 (3)	0.6997 (2)	0.0392 (6)	
C8	0.6254 (4)	0.0747 (3)	0.6550 (2)	0.0416 (6)	
C9	0.8197 (4)	0.0410 (3)	0.5729 (2)	0.0366 (5)	
C10	0.9840 (4)	0.1665 (3)	0.5176 (2)	0.0424 (6)	
H10A	0.9741	0.2781	0.5300	0.051*	
C11	1.1621 (4)	0.1265 (3)	0.4442 (2)	0.0420 (6)	
H11A	1.2700	0.2117	0.4063	0.063*	

### Atomic displacement parameters (Å^2^)

**Table:**

	*U*^11^	*U*^22^	*U*^33^	*U*^12^	*U*^13^	*U*^23^
S1	0.0734 (5)	0.0418 (4)	0.0480 (4)	0.0108 (3)	0.0225 (3)	−0.0049 (3)
N1	0.0520 (13)	0.0393 (11)	0.0402 (12)	0.0059 (9)	0.0153 (10)	−0.0063 (9)
N2	0.0425 (11)	0.0406 (11)	0.0337 (11)	0.0043 (9)	0.0123 (9)	−0.0046 (9)
O1	0.0729 (13)	0.0485 (11)	0.0597 (12)	0.0131 (9)	0.0335 (10)	0.0042 (10)
C1	0.0528 (16)	0.0529 (16)	0.0418 (15)	0.0031 (13)	0.0062 (12)	−0.0049 (12)
C2	0.0677 (19)	0.0633 (19)	0.0397 (15)	−0.0032 (15)	0.0129 (13)	−0.0097 (13)
C3	0.0584 (18)	0.0592 (18)	0.0540 (18)	−0.0055 (14)	0.0247 (14)	−0.0205 (14)
C4	0.0433 (16)	0.0655 (19)	0.067 (2)	0.0032 (13)	0.0102 (14)	−0.0221 (15)
C5	0.0451 (15)	0.0641 (18)	0.0419 (15)	0.0022 (13)	0.0032 (12)	−0.0137 (13)
C6	0.0410 (14)	0.0391 (14)	0.0430 (14)	−0.0015 (11)	0.0105 (11)	−0.0110 (11)
C7	0.0384 (13)	0.0445 (14)	0.0346 (13)	0.0039 (11)	0.0027 (10)	−0.0106 (11)
C8	0.0419 (14)	0.0419 (14)	0.0386 (14)	0.0050 (11)	0.0056 (11)	−0.0070 (12)
C9	0.0368 (13)	0.0406 (13)	0.0318 (12)	0.0052 (10)	−0.0009 (10)	−0.0079 (10)
C10	0.0437 (14)	0.0376 (14)	0.0462 (15)	0.0032 (11)	0.0040 (11)	−0.0124 (11)
C11	0.0391 (13)	0.0401 (14)	0.0439 (14)	−0.0001 (10)	0.0086 (11)	−0.0068 (11)

### Geometric parameters (Å, °)

**Table:**

S1—C7	1.667 (2)	C3—C4	1.372 (4)
N1—C7	1.327 (3)	C3—H3A	0.93
N1—C6	1.433 (3)	C4—C5	1.383 (3)
N1—H1A	0.86	C4—H4A	0.93
N2—C8	1.373 (3)	C5—C6	1.383 (3)
N2—C7	1.396 (3)	C5—H5A	0.93
N2—H2A	0.86	C8—C9	1.495 (3)
O1—C8	1.220 (3)	C9—C10	1.388 (3)
C1—C6	1.376 (4)	C9—C11^i^	1.390 (3)
C1—C2	1.389 (4)	C10—C11	1.382 (3)
C1—H1B	0.93	C10—H10A	0.93
C2—C3	1.361 (4)	C11—C9^i^	1.390 (3)
C2—H2B	0.93	C11—H11A	0.93
			
C7—N1—C6	126.8 (2)	C4—C5—H5A	120.4
C7—N1—H1A	116.6	C1—C6—C5	120.3 (2)
C6—N1—H1A	116.6	C1—C6—N1	118.3 (2)
C8—N2—C7	128.3 (2)	C5—C6—N1	121.3 (2)
C8—N2—H2A	115.8	N1—C7—N2	115.9 (2)
C7—N2—H2A	115.8	N1—C7—S1	125.62 (18)
C6—C1—C2	119.5 (3)	N2—C7—S1	118.42 (17)
C6—C1—H1B	120.2	O1—C8—N2	122.6 (2)
C2—C1—H1B	120.2	O1—C8—C9	121.2 (2)
C3—C2—C1	120.4 (3)	N2—C8—C9	116.2 (2)
C3—C2—H2B	119.8	C10—C9—C11^i^	119.5 (2)
C1—C2—H2B	119.8	C10—C9—C8	123.1 (2)
C2—C3—C4	120.1 (2)	C11^i^—C9—C8	117.4 (2)
C2—C3—H3A	119.9	C11—C10—C9	120.3 (2)
C4—C3—H3A	119.9	C11—C10—H10A	119.8
C3—C4—C5	120.6 (3)	C9—C10—H10A	119.8
C3—C4—H4A	119.7	C10—C11—C9^i^	120.1 (2)
C5—C4—H4A	119.7	C10—C11—H11A	119.9
C6—C5—C4	119.2 (3)	C9^i^—C11—H11A	119.9
C6—C5—H5A	120.4		

Symmetry codes: (i) −*x*+2, −*y*, −*z*+1.

### Hydrogen-bond geometry (Å, °)

**Table:**

*D*—H···*A*	*D*—H	H···*A*	*D*···*A*	*D*—H···*A*
N1—H1A···O1	0.86	1.94	2.644 (3)	138
N2—H2A···S1^ii^	0.86	2.62	3.446 (3)	160

Symmetry codes:  (ii) −*x*+1, −*y*+1, −*z*+1.
